# Progress in Composite Hydrogels and Scaffolds Enriched with Icariin for Osteochondral Defect Healing

**DOI:** 10.3390/gels8100648

**Published:** 2022-10-12

**Authors:** Elena Iulia Oprita, Andreea Iosageanu, Oana Craciunescu

**Affiliations:** National Institute of R&D for Biological Sciences, 296, Splaiul Independentei, 060031 Bucharest, Romania

**Keywords:** hydrogel, flavonoids, cartilage, osteoarthritis, osteochondral defect, bone morphogenetic proteins

## Abstract

Osteochondral structure reconstruction by tissue engineering, a challenge in regenerative medicine, requires a scaffold that ensures both articular cartilage and subchondral bone remodeling. Functional hydrogels and scaffolds present a strategy for the controlled delivery of signaling molecules (growth factors and therapeutic drugs) and are considered a promising therapeutic approach. Icariin is a pharmacologically-active small molecule of prenylated flavonol glycoside and the main bioactive flavonoid isolated from *Epimedium* spp. The in vitro and in vivo testing of icariin showed chondrogenic and ostseoinductive effects, comparable to bone morphogenetic proteins, and suggested its use as an alternative to growth factors, representing a low-cost, promising approach for osteochondral regeneration. This paper reviews the complex structure of the osteochondral tissue, underlining the main aspects of osteochondral defects and those specifically occurring in osteoarthritis. The significance of icariin’s structure and the extraction methods were emphasized. Studies revealing the valuable chondrogenic and osteogenic effects of icariin for osteochondral restoration were also reviewed. The review highlighted th recent state-of-the-art related to hydrogels and scaffolds enriched with icariin developed as biocompatible materials for osteochondral regeneration strategies.

## 1. Introduction

Osteochondral defects represent usual clinical issues for orthopedic surgeons around the world. They involve both articular cartilage and underlying subchondral bones and are the result of traumatic injuries, aging, and degradative diseases, such as osteoarthritis (OA), osteochondritis dissecans, or osteonecrosis [1,2].

OA is the most encountered chronic joint disorder worldwide with a multifactorial etiology and a major cause of pain and disability in the elderly [3]. It generally affects the knee, hip, and hand, but also acts on any synovial joint, including the joints of the spine [4]. OA is characterized by chronic pain and joint stiffness caused by progressive degradation of the articular cartilage, synovial inflammation, abnormal remodeling of subchondral bone, and calcification of ligaments [5]. All of these lead to loss of mobility and functional decline of the affected joints, lowering the quality of life and, in the worst scenario, causing disability.

The cellular and molecular mechanisms of OA pathogenesis are not fully understood, but it is certain that OA manifestations are caused by an imbalance between cartilage degradation and repair processes, in which the extracellular matrix (ECM) remodeling enzymes are involved [6,7]. Advancing age is the most important risk factor of OA [8]. Other major risk factors are genetic susceptibility, sex, obesity, metabolic diseases, joint injuries, mechanical stress, occupation, and diet [9,10]. Commonly, OA affects people over the age of 40, and the risk of disease increases with age, reaching 40% of people over the age of 70 suffering from OA [6,11]. In 2017, 303 million people were globally affected by OA [12] and, according to the most recent report from 2019, the number of people suffering from this condition exceeded 500 million (prevalence of 6.5%) [13]. WHO estimates that the number of people affected by OA in the U.S. will increase from 30 million (9% prevalence) to 67 million by the year 2030 [8] and it will reach 130 million by the year 2050 [14]. In Europe, the prevalence of OA varies from 2.8% in Romania to over 12% in the United Kingdom and 18% in Hungary [14,15] and it is estimated that 20% of the population of Western Europe will suffer from this disease by 2023 [16]. In Australia, the prevalence of OA is 9.3% [17]. More recent data show that the global incidence of knee OA is 3.5 times higher than hand OA and 3 times higher than hip OA in patients around the age of 75 [18]. In addition to the suffering and social impacts, OA is also a major economic burden. In the U.S., the yearly estimates of OA spending are over USD 185 billion [19], with half of the direct costs going to patients’ arthritis drugs, mostly pain-related medications [14].

The management of OA symptoms involves non-pharmacological, pharmacological, and surgical treatments to reduce pain and slow the progression of the disease, the treatment being symptomatic, and not curative [20]. Non-pharmacological treatment mainly consisting of physical therapy is often associated with pharmacological treatment that involves the administration of analgesics and/or intra-articular administration of hyaluronic acid, in which case short-term effects are recorded [19,21]. In more severe cases, surgery is performed by replacing the joints, but this form of treatment is not completely curative because of the persistence of postoperative symptoms, which are present in approximately 20% of the patients. Both the European Society for Clinical and Economic Aspects of Osteoporosis, Osteoarthritis and Musculoskeletal Diseases (ESCEO) and the Osteoarthritis Research Society International (OARSI) recommend non-surgical treatment to patients suffering from knee OA [22].

Natural compounds, such as flavonoids, are increasingly explored for their therapeutic potential in cartilage and bone health. Icariin (ICA) is described as a bioactive flavonoid due to its multiple pharmacological effects, such as osteoprotective, anti-osteoporosis, anti-inflammatory, and antioxidative effects, and a positive effect on the reproductive system [16,23,24]. In addition to its biological activities that support bone health, ICA improves cardiovascular function, regulates hormones, enhances the immune response, and has anti-hepatotoxic, antiviral, and anticancer activity [25,26,27,28].

Recent studies have shown the advantage of using ICA in osteochondral regeneration due to both osteogenic and chondrogenic effect, compared to other flavonoids, such as naringin and kaempferol, which presented only osteogenic effects [23]. Reports on another flavonoid, quercetin, indicated its osteogenic and chondrogenic effects, but evidence on cartilage regeneration and the mechanisms of action were limited [29].

This review presents the osteochondral tissue structure, underlining the basic aspects of osteochondral defects and those specific to OA, the ICA structure, and extraction methods, emphasizing the importance of ICA chondrogenic, and osteogenic effect in osteochondral restoration. Moreover, the review highlighted the recent state-of-the-art related to hydrogels and scaffolds enriched with ICA and their use as biocompatible materials for osteochondral regeneration strategies, which must take into account the healing of subchondral bone and articular cartilage, as well as the joint interface. To our knowledge, this is the first paper that presents the effect and the importance of ICA-incorporated biomaterials in the field of osteochondral engineering.

## 2. Osteochondral Tissue and Associated Defects

### 2.1. Osteochondral Tissue

Osteochondral tissue regeneration represents a challenge of regenerative medicine due to its structural complexity, layered architecture, and biomechanical properties. Hierarchically, the osteochondral tissue consists of articular cartilage, osteochondral interface, and the underlying subchondral bone.

***Human adult articular cartilage*** (hyaline cartilage) (2–4 mm thick) is void of blood vessels, nerves, or lymphatics and has a specific structure and composition adapted to its function of load-bearing surface and lubrication of synovial joints. The structural organization of articular cartilage plays a central role in modulating the biomechanical function, according to cartilage thickness, chondrocyte-mediated matrix deposition, and remodeling or tissue loading parameters [30]. Histologically, the human adult articular cartilage is a multiphase connective tissue composed of structurally and biochemically distinct zones: the superficial, intermediate and deep zone [31,32,33]. Each zone has its own: (i) ECM composition, consisting mainly of collagen (COL) type II, other non-collagenous proteins, proteoglycans, and water; (ii) COL orientation; and (iii) chondrocytes’ phenotype, morphology, density, and rate of metabolic activity [30,34].

The ECM secreted by chondrocytes is heterogeneous and has been divided into three distinct regions: the pericellular, territorial and interterritorial region, based on proximity to the cells, composition, and COL fibril diameter and organization. Each matrix zone is characterized by different COL types and the expression of distinct molecules [35,36]. Thus, the pericellular zone is a thin layer rich in COL type II, VI, and IX and proteoglycans, such as aggrecan, hyaluronan, and decorin, placed next to cell membrane receptors, as the integrins. The territorial zone contains COL type VI, non-collagenous molecules, such as matrilins 1 and 3, and the small proteoglycans, biglycan and decorin. The interterritorial zone is composed of COL types II, IX, and XI, non-collagenous molecules, such as matrilin 3, aspirin, and cartilage oligomeric protein, and proteoglycans, such as heparan sulfate, fibromodulin and decorin [37]. The depth-dependent composition, organization, and structure are responsible for the unique biomechanical properties of the articular cartilage, such as tensile strength, flexibility, and load-bearing ability [38,39]. Thus, the recorded values of the compressive modulus of its superficial, middle, and deep zone were 0.079, 2.1, and 320 MPa, respectively, indicating notable differences in the stiffness of this tissue [40].

Chondrocytes, the only cellular component of articular cartilage have different morphologies, varying from more flattened at the surface to more rounded and larger in the deeper zones. They live within the cartilage matrix at low oxygen tension, ranging from 10% at the surface to less than 1% in the deep zones [32,41].

***The superficial (tangential) zone*** (10–20%) has an ECM consisting of COL fibrils of 30–35 nm in diameter (86%), tightly packed and oriented parallel to the articular surface, mainly represented by COL types II (60–70%) and IX, and minimal proteoglycan content, such as aggrecan (5–15%) [2,31,42]. The synovial fluid present in the cartilage overlaying zone consists of a high proportion of water (70–84%), hyaluronic acid, and lubricin, a splice form of proteoglycan 4, enabling the gliding surface [37,41,43].

The chondrocytes in this zone exhibit small and circular morphology (flattened ellipsoid) and lie parallel to the surface [41,43] and show distinct spatial patterns as single cells, pairs, clusters, or strings, depending on the joint type [44]. They also present the properties of mesenchymal stem cells [45]. The superficial zone is covered by lamina splendens, a clear film with small COL fibrils, which have a parallel orientation to the articular surface and a cellular layer of flattened chondrocytes [46].

This thin zone acts as a barrier regulating the diffusion transport of nutrients and oxygen to the underlying cartilage structure, as well as the ingress and egress of large biomolecules [2]. It also has good tensile strength providing high resistance to strong shear forces, due to well-organized COL fibrils. Still, disruption of the superficial zone results in the alteration of the cartilage’s mechanical properties and is the first to show degenerative changes, contributing to the development of OA [46].

***Intermediate (middle) zone*** (40–60%) contains round chondrocytes distributed among randomly oriented COL fibrils and the highest proteoglycans concentration. In the middle zone, chondrocytes synthesize high amounts of aggrecan and there are also variable amounts of small proteoglycans [41]. Under normal, low turnover conditions, chondrocytes resting in the unstressed steady state maintain the synthesis of proteoglycans and other non-collagenous molecules [47]. There is very little turnover of COL type II [48]. Functionally, the middle zone is the first line of resistance to compressive forces [35].

***Deep (radial) zone*** (20–30%) presents round or ellipsoid chondrocytes, which are packed in columns parallel to the organized COL fibers and show 10-fold higher synthesis activity than the cells in the superficial zone, while they have only twice as much surface area and volume. In the deep zone, COL fibrils (67%) have a larger diameter of up to 40–80 nm and are oriented perpendicular to the articular surface, while the proteoglycans (20%) and water (40–60%) content is lower than in the superficial and intermediate zones [31].

***The cartilage-bone interface*** represents a network hub anatomically connecting the articular cartilage and the subchondral bone. The complex composition and morphological characteristics of this interface are required to make the transition between significantly different physiology and biomechanical properties of the cartilage and those of the subchondral bone [32].

Tidemark (approximately 3 µm in thickness), a complex three-dimensional structure visible between the non-calcified deep zone and the underlying calcified cartilage, anatomically divides the cartilage from the subchondral bone [49]. The wavy tidemark composed of matrix vesicles serves to COL fibrils attachment, allows nutrient diffusion through small gaps located within the structure, and prevents the invasion of vasculature and nerves into the articular cartilage [31,32].

Calcified cartilage is the main component of the osteochondral interface, bridging the unmineralized articular cartilage to the subchondral bone. It contains hypertrophic chondrocytes, has a unique ECM composition with a considerable amount of hydroxyapatite, COL type X (vertically oriented fibrils), is void of proteoglycans [41,49], and is connected to the subchondral bone through interdigitations, which assist in transforming the articulation shear stress into tensile and compressive stress [31]. The calcified zone has lower permeability than the articular cartilage, which allows the migration of molecules smaller than 500 Da from the subchondral bone to the cartilage in both directions [42]. Moreover, the calcified zone has vascularization and innervations originating from the subchondral bone in association with advanced age [41].

The cement line represents the lower boundary, marking the COL type and fiber orientation changes [42].

***Subchondral bone***, an important part of subchondral tissue, consists of the subchondral bone plate and the subarticular spongiosa and is separated from the calcified cartilage by the cement line. The subchondral bone maintains the shape of the joint bone and provides a suitable mechanical and biological environment for cartilage development and differentiation [50]. The subchondral region has characteristic anatomy, but is variable in thickness, density, and composition of the subchondral bone plate, the contour of the tidemark, and cement line, and the number and types of channels penetrating the calcified cartilage [51]. The main components of the subchondral bone include: HA, COL types I and X, fibronectin, and laminin [40]. Moreover, the subchondral bone has powerful compressive strength and strong stiffness provided by considerable amounts of hydroxyapatite and COL type I fibers, high compressive modulus (5.7 GPa), and low elasticity [32,40].

### 2.2. Osteochondral Defects: Basic Aspects and Restoration

**Figure 1:**
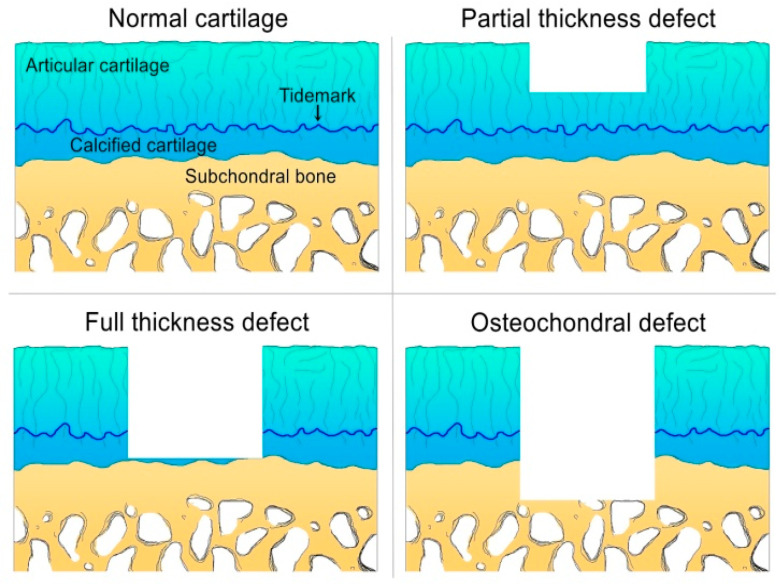
Types of cartilage defects. Partial thickness defect develops only in the articular cartilage. In full-thickness defect, the subchondral bone plate is exposed, but not disturbed. The osteochondral defect develops in the cartilage and subchondral bone.

The defects affecting only the articular cartilage layer or chondral defects can be divided into the partial thickness and full-thickness defects, according to their depths, while osteochondral defects extend deep into the subchondral bone (Figure 1).

Partial thickness defects of the articular cartilage do not reach down to the subchondral bone and fail to heal spontaneously, due to the fact that they do not have access to the progenitor cells from the bone marrow space [43,52]. In mature tissue, a limited repair process takes place at the defect situs, immediately after the injury.

Full-thickness defects are lesions that affect the areas up to the subchondral bone comprising the articular cartilage, tidemark, and calcified cartilage [52].

Osteochondral defects are lesions or disruptions of the articular cartilage and subchondral bone that occur after an acute traumatic injury of the joint or an underlying disorder of the bone appeared on specific weight-bearing sites, at the end of the thighbone, shinbone, and the back of the kneecap [53]. In contrast to the chondral defects that have poor healing capacity, the small osteochondral defects present healing capacity by recruitment of the non-differentiated bone marrow mesenchymal stem cells from the damaged site [54].

Osteochondral regeneration involves simultaneous renewal of three tissues with different mechanical and biological properties: the articular cartilage, osteochondral interface (the functional calcified layer), and osteochondral bone. Due to the weak regeneration capacity of the articular cartilage, two major problems need to be taken into account for its repair: (1) to fill the defect with a tissue that has the same mechanical properties as articular cartilage and (2) to promote successful integration between the repair tissue and the host/native articular cartilage [55]. Calcified zone regeneration is essential for integrative and functional osteochondral repair [56]. Despite the remarkable advances in the field of osteochondral/cartilage tissue engineering through marrow stimulation, autologous chondrocytes implantation, and osteochondral autografts and allografts, the restoration of osteochondral interface and full-thickness articular cartilage defects remain challenging [57]. Among the proposed therapies, osteochondral tissue engineering has shown considerable promise using multiple approaches based on cells, scaffolds and signaling molecules. Ideally, osteochondral structure reconstruction by tissue engineering requires a scaffold that provides both articular cartilage and subchondral bone. Along with scaffold design, effective inductors are needed to efficiently synthesize the osteochondral ECM. Furthermore, functional scaffolds with controlled delivery of signaling molecules (growth factors, therapeutic drugs, and genes) are considered a promising therapeutic approach [58]. Studies have reported that certain growth factors (bone morphogenetic protein-2 (BMP-2), BMP-7, insulin-like growth factor-1 (IGF-1), fibroblast growth factor-2 (FGF-2) can support the maturation of cartilage, while others (IGF-1, IGF-2, platelet-derived growth factor, transforming growth factor-beta (TGF-β), BMP-2, BMP-4, BMP-6, and BMP-7) can induce osteogenic differentiation [32]. The signaling molecules induce ECM formation within the osteochondral tissue, while growth factors and cytokines residing in the scaffolds are used to stimulate the formation of osteochondral structural constituents.

## 3. ICA—Trigger for Osteochondral Regeneration

ICA has been used in traditional Chinese medicine for over 2000 years to cure cartilage- and bone-related disorders, such as OA and osteoporosis (bone microstructure degeneration, bone mass reduction, and bone fractures) [24,59,60] and is widely used in China, Japan and Korea as an antirheumatic drug [61].

Studies have reported that ICA promotes bone formation, can alleviate bone mass loss, improves the degree of bone mineralization, and can prevent estrogen deficiency-induced bone fractures by activating the estrogen receptors. Moreover, ICA inhibits osteoclast differentiation, and reduces motility and bone resorption, being a potential bone strengthen inductor [24].

Vascularization is important in bone formation; some research studies have reported that ICA promotes endothelial cell proliferation and tubulogenesis, could protect vascular endothelial cells with an anti-apoptosis effect, and could activate angiogenesis [24].

### 3.1. ICA Structure

**Figure 2:**
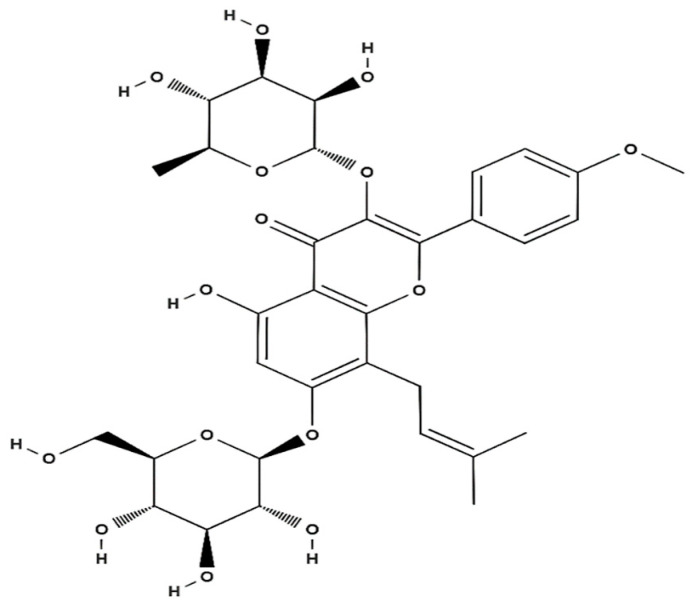
Chemical structure of icariin.

ICA is isolated from the dried leaves of the medicinal plant “yinyanghuo” (*Epimedium* spp.), as the main bioactive flavonoid, representing no less than 0.5% from 5.0% total flavonoid content. ICA or 2-(4′-methoxylphenyl)-3-rhamnosido-5-hydroxyl-7-glucosido-8-(3′-methyl-2-butylenyl)-4-chromanone is a pharmacologically active small molecule of prenylated flavonol glycoside (Figure 2). Its molecular formula is C_33_H_40_O_15_, it has a molecular weight of 676.67 g/mol and a melting point of 231~232 °C [62,63].

Pharmacokinetic studies have shown that it is not the original form of ICA that is responsible for these biological effects, but its metabolites. Qian et al. (2012) [64] have found 19 different metabolites in rat plasma after injection of ICA, including icariside I and II, icaritin, desmethyl icaritin, icaritin-3,7-di-O-glucuronide, and icaritin-3-O-rhamnose-7-O-glucuronide.

### 3.2. ICA Extraction Methods

Traditional methods of ICA extraction use polar solvents, such as ethyl acetate and ethanol, because it is insoluble in non-polar solvents, such as chloroform, ether, and benzene [62]. The use of chemical solvents for the extraction of compounds intended for medical purposes can be resource-consuming and also harmful to human health. Thus, new adsorptive materials with specific selectivity to ICA were synthesized to improve ICA extraction, as an alternative to traditional methods. Eutectic solvents are a new generation of solvents characterized by easy preparation, solute stabilization, good biodegradability, and cost-effectiveness, which can be classified into ionic liquids and deep eutectic solvents. Therefore, deep eutectic solvents have been proposed for the extraction of ICA, being safer for humans and also for the environment [65]. Recently, Wang et al. (2020) [66] have published a comparative study analyzing the yield of ICA extraction from *Epimedium pubescens Maxim* by an ultrasonic-assisted method using traditional solvents, such as methanol, 70% ethanol, 50% ethanol and water, on the one hand, and deep eutectic solvents with choline chloride as the first component, and lactic acid, ethylene glycol, glycerol and 1,2-propanediol as the second component, in different molar ratios, on the other hand. The results have shown that the highest yield (3.25 mg/g) was recorded for ICA extraction in 50% ethanol, while the second highest extraction yield (3.12 mg/g) was found for a mixture consisting of choline chloride:lactic acid mixture (1:2 ratio), indicating the successful use of the latter as an eco-friendly alternative to traditional solvents [66]. However, another team reported that the extraction efficiency of the L-proline:ethylene glycol mixture (1:4 ratio) was better, compared to the choline chloride:lactic acid mixture (1:2 ratio) and 50% ethanol [65].

In terms of ICA extraction techniques applied to the plant material, the classical one uses the extraction by stirring, but improved yields are obtained by heat refluxing, pressurized microwave-assisted, atmospheric pressure microwave-assisted, and ultrasonic-assisted techniques [67]. Wang et al. (2020) [66] compared the stirring, heat refluxing, and ultrasonic-assisted extraction methods using deep eutectic solvents with low volatility and high viscosity. As expected, the extraction of ICA by the stirring method had the lowest yield, and the addition of heat did not improve the extraction yield. The extraction of ICA through the ultrasonic-assisted method registered the best yield, but was not significantly different from that of heat refluxing. In the case of using 40% ethanol for ICA extraction, the best extraction yield was reported by another team for the pressurized microwave-assisted technique, followed by heat refluxing and atmospheric pressure microwave-assisted techniques, while the lowest yield was obtained for the ultrasonic-assisted technique [67]. The difference between the extraction efficiency in these two studies was most likely due to the use of different extraction solvents.

### 3.3. ICA Effect on Osteochondral Regeneration

Although long used in traditional medicine, scientific investigations on the chondroprotective and osteoprotective effects of ICA were performed in the last period. Some reports showed that ICA might be a potential accelerator of chondrogenesis, modulating the proliferation of chondrocytes and reducing ECM degradation [16,68].

**Figure 3:**
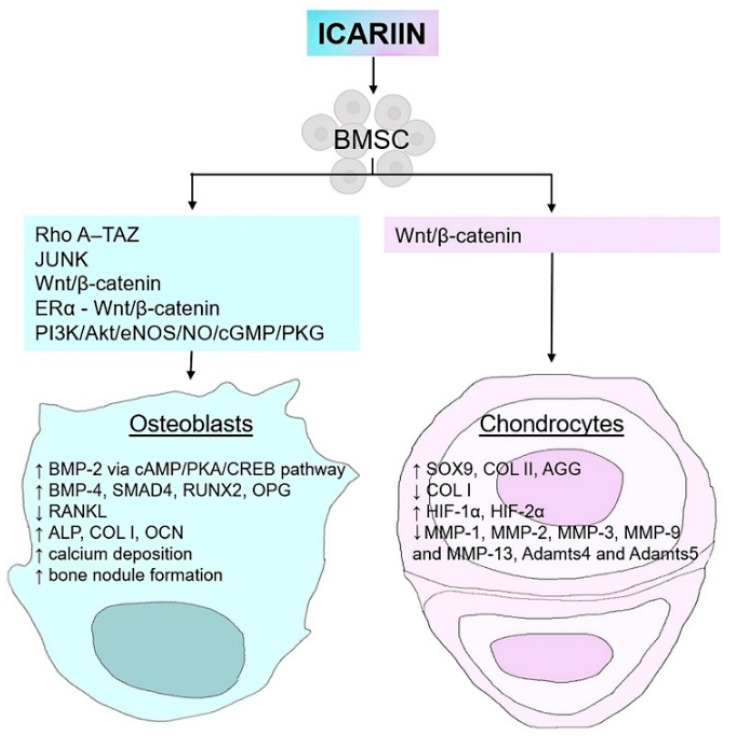
Icariin’s effect on bone marrow stem cells (BMSCs) as a promoter of bone formation and a potential accelerator of chondrogenesis via different signaling pathways. Icariin exerts an osteogenic effect by promoting the expression of osteoblastic specific genes, bone morphogenetic proteins (BMP-2, BMP-4), SMAD4, RUNX2, osteoprotegerin (OPG), alkaline phosphatase (ALP), collagen type I (COL I), osteocalcin (OCN) and the downregulation of the receptor activator of nuclear factor kappa-B ligand (RANKL). Icariin also upregulates calcium deposition and bone nodule formation. Its chondrogenic effect is due to the upregulated expression of SRY-Box transcription factor 9 (SOX9), collagen type II (COL II), aggrecan (AGG), and the downregulation of COL I. It also serves as an activator of hypoxia-inducible factors (HIF-1α and HIF-2α) and several metalloproteinases (MMP) and a disintegrin and metalloproteinase with thrombospondin motifs (ADAMTS) in chondrocytes.

Previous studies suggested that high concentration of ICA promoted ECM synthesis (glycosaminoglycans and COL), upregulated the expression of SRY-type high mobility group box 9 (SOX9) (early chondrogenic marker), COL type II and aggrecan genes [69] and downregulated the expression of COL type I gene in the 2D culture of chondrocytes [70] (Figure 3).

ICA has demonstrated great in vitro potential to promote bone formation and suppress bone resorption. A significant osteogenic effect of ICA was observed in human bone mesenchymal stem cells, rat bone marrow stromal cells, primary osteoblasts (human, rat, and mouse), and osteoblast-like cells (UMR 106 cells) [71,72].

ICA has revealed its action as a potent bone anabolic agent, which is comparable to BMP-2, enhancing the proliferation and osteogenic differentiation of MC3T3-E1 cells [73,74,75]. Hsieh et al. (2010) [76] have examined the molecular mechanisms of ICA in regulating osteoblast metabolism, showing that, at a concentration as low as 10^−8^ M ICA, cell proliferation and matrix mineralization reached maximum values and promoted NO synthesis. Moreover, ICA treatment upregulated the gene expression of BMP-2 by activating the cAMP/PKA/CREB signaling pathway [26], BMP-4, SMAD4, runt-related transcription factor (RUNX2), and osteoprotegerin and downregulated the gene expression of receptor activator of nuclear factor kappa-B ligand (RANKL) [77,78]. Cell viability and proliferation, osteogenic differentiation markers (alkaline phosphatase, COL type I, osteocalcin), calcium deposition, and bone nodule formation increased in the presence of ICA in a dose-dependent manner [79].

ICA promoted bone marrow stem cell (BMSC) osteogenesis via different signaling pathways, such as RhoA-TAZ, JNK, Wnt/*β*-catenin, ER*α*-Wnt/*β*-catenin, and PI3K/Akt/eNOS/NO/cGMP/PKG [80]. Moreover, it was shown that ICA inhibited the expression of the proteins related to the fusion and formation of osteoclasts, suppressed the activity of tartrate-resistant acid phosphatase (TRAP) and the expression of matrix metalloproteinase-9 (MMP-9) [81]. A report has indicated that ICA had an osteoinductive effect in vivo through the process of endochondral ossification [82].

A recent study revealed the capacity of ICA to exert anti-osteoporotic and anti-inflammatory effects in OA by preventing inflammation and chondrocytes apoptosis through activation of autophagy via inhibiting nuclear factor kappa-B (NF-kB) signaling pathway [83]. Wang et al. (2020) [84] demonstrated that ICA increased the vitality of chondrocytes by suppressing inflammation through the inhibition of the NF-kB/HIF-2α signaling pathway. Moreover, experiments in interleukin-1β-stimulated chondrosarcoma cells have shown that ICA exerted a chondroprotective effect through the inhibition of MMP-1, MMP-3, and MMP-13 and the suppression of osteoprotegerin, RANKL and the receptor activator of nuclear factor kappa-B (RANK) system via MAPK pathway [85,86].

An important role in inflammation is played by hypoxia-inducible factors (HIFs) family members, HIF-1α and HIF-2α, consisting of α- and β- subunits, which are highly expressed in the OA cartilage of mice and humans. ICA could upregulate HIF-1α expression, as a key mediator of chondrocyte response to oxygen fluctuations during cartilage development and repair and maintain the chondrocyte phenotype [87,88]. The effect of ICA on HIF-2α is yet unclear. ICA has inhibited the expression of NF-kB and HIF-2α in mice bone defect, but it showed activation of HIF-2α, MMP-9, and expression of disintegrin and metalloproteinase with thrombospondin motifs 5 (ADAMTS5), as key targets of the NF-kB/HIF-2α signaling pathway, in TNF-α-treated ADTC5 chondrocytes [84,89].

Zu et al. (2019) [83] have demonstrated that ICA reduced LPS-induced pyroptosis and the inhibition of COL formation through suppression of the NLRP3 inflammasome-mediated caspase-1 signaling pathway. The in vivo effect was further confirmed in the rat OA model by ICA inhibition of NLRP3-mediated pyroptosis. Thus, ICA might play a therapeutic role in OA treatment [83].

Taken together, all the mentioned characteristics of ICA demonstrated by studies related to in vitro and in vivo cell proliferation, osteogenic and chondrogenic differentiation, and the use of ICA alone or in combination with other molecules by direct administration or integrated into composite hydrogels and scaffolds, represent a very promising and low-cost approach for osteochondral regeneration and regenerative medicine.

## 4. Hydrogels and Scaffolds Enriched with ICA for Osteochondral Tissue Engineering

**Figure 4:**
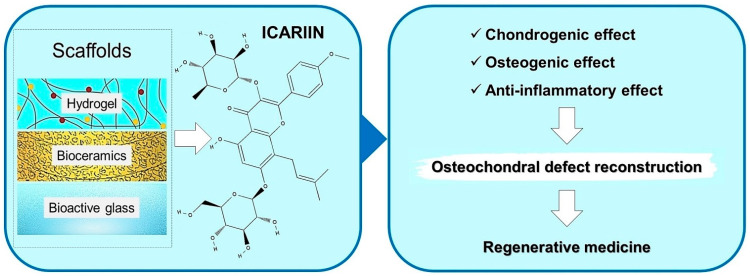
Types of functional composite biomaterials conditioned as hydrogel, containing bioceramics or bioglasses for controlled delivery of icariin are a promising therapeutic approach in osteochondral defect reconstruction.

The damaged cartilage has a limited capacity for self-healing due to the lack of blood vessels, nerves, lymph supplies, and the poor differentiating and migratory abilities of chondrocytes. Clinically, several methods used to treat osteochondral defects, such as debridement and bone marrow stimulation techniques or osteochondral grafts (allograft and autografts) did not provide satisfactory outcomes [82]. Tissue engineering based on biodegradable hydrogels and scaffolds made of natural materials, synthetic polymers, ceramic materials, or bioglasses with or without biotic factors, such as growth factors (TGF-β1, BMP-2) and cells (chondrocytes and mesenchymal stem cells) provides the tools for the reconstruction of osteochondral defects [40]. ICA can be steadily and locally released from different biomaterials, thus becoming an attractive candidate for osteochondral tissue engineering (Figure 4).

Jia et al. (2015) [63] prepared ICA molecularly imprinted polymers by precipitation polymerization using acrylamide as functional monomer, ethylene glycol dimethacrylate as cross-linker, and methanol:acetonitrile mixture (3:1, *v*/*v*) as the reaction solvent, in a molar ratio of template molecule:functional monomer:cross-linker of 1:6:80. An innovative therapy using autologous conditioned serum was proposed as being able to improve tissue regeneration and to reduce the degenerative mechanisms of OA [23,53]. Moreover, ICA-conditioned serum and ICA-conditioned serum combined with chitosan had a positive effect on primary rabbit chondrocyte proliferation and it was also demonstrated the regeneration of cartilage in rabbit osteochondral defect models [53]. Moreover, it was observed that the ICA-conditioned serum and hyaluronic acid treatment of rabbit femoral condyle have regenerated the cartilage and subchondral bone [23]. In vitro and in vivo studies have investigated the potential of several cytocompatible, biomimetic biomaterials containing ICA and natural polymers (fibrin, silk fibrin, hyaluronan, alginate, chitosan, COL), synthetic polymers (polylactic acid, poly(lactic-co-glycolic acid), polyhydroxyethylmethacrylate, polycaprolactone), ceramics (hydroxyapatite, tricalcium phosphate), conditioned as composite hydrogels and scaffolds for cartilage repair and bone regeneration in osteochondral defects [82]. ICA was combined with calcium phosphate cement tablets [73], β-tricalcium phosphate disks [90], 3D complex alginate hydrogels [87], gelatin/hyaluronic acid composite microspheres [91], porous poly(3-hydroxybutyrate-co-3-hydroxyvalerate) scaffolds [36], chitosan/hydroxyapatite scaffolds [92], nanodiamonds [93] and, more recently, with bioactive glasses [94]. Reiter et al. (2019) [95] have fabricated 3D sponge-like scaffolds based on 45S5 bioactive glass coated with gelatin, as a suitable vehicle for ICA delivery (Table 1).

Yuan et al. (2015) [96] have shown that ICA might be a substitute for growth factors in cartilage tissue engineering when used through chemical conjugation to a hyaluronic acid/COL hydrogel. The presence of ICA and hyaluronic acid macromolecules had no significant effect on the mechanical properties and degradation of the composite hydrogel. The prepared hydrogel maintained the seeded chondrocytes’ morphology and promoted the biosynthesis of the cartilage matrix. It was reported an increase of SOX9, aggrecan, and COL type II gene expression in chondrocytes and the production of glycosaminoglycans and COL type II in the hydrogel. Thus, the formation of new cartilage in ICA-loaded hydrogel was better than that in hydrogel without ICA, underlining the positive role of ICA chemical conjugation to the polymeric components [96].

The same hydrogel seeded with mesenchymal stem cells showed that conjugated ICA can be gradually released and had the ability to promote both cartilage and bone formation, depending on the used inductive medium [97]. Thus, under cartilage induction conditions, the cells seeded in ICA-loaded hydrogel showed chondrocyte-like morphology, upregulation of the expression of cartilage-related genes, and increase of the secretion of cartilage matrix in the hydrogel, while in the bone induction microenvironment, the seeded cells showed osteoblast morphology, the deposition of calcium, and a significant increase of the expressions of bone-related genes and proteins. These results have suggested enhanced osteochondral defect repair ability of the hyaluronic acid-COL hydrogel after ICA chemical conjugation [97].

Gorji et al. (2020) [99] have prepared novel delivery systems of fibrin-ICA nanoparticles loaded in poly(lactic-co-glycolic acid) polymeric biomaterials for the chondrogenic effect on human adipose tissue stem cells. The in vivo anabolic effect of ICA was confirmed in a mouse calvarial defect model [73]. The study has shown significant new bone formation in C57BL/6N mice, at 4 weeks after transplantation with ICA-calcium phosphate cement tablets, compared to tablets without ICA. After 6 weeks, the new bone thickness increased and blood vessel formation was observed [73]. Rabbit chondrocytes were embedded within an in vitro-engineered ICA/COL I hydrogel used for the restoration of adult rabbit osteochondral defect [82]. It was shown that ICA upregulated the expression of aggrecan, SOX9, and COL type II in seeded chondrocytes and, also, accelerated the formation of chondroid tissue in the hydrogel. It improved the restoration of the osteochondral defect in the adult rabbit model and enhanced the integration of newly-formed cartilage with subchondral bone [82]. Zhao et al. (2019) [100] loaded ICA onto poly(lactic-co-glycolic acid) fibers by electrospinning to prepare a composite scaffold with high hydrophilicity and good biocompatibility, showing a slow and steady release of ICA. In models of rabbit OA, the composite scaffold promoted the synthesis of ECM components (COL type II, aggrecan), maintained the functional morphology of the articular cartilage, and inhibited the resorption of subchondral bone trabeculae, demonstrating its therapeutic potential to inhibit OA progression. A new core-shell scaffold was fabricated using the polymers COL, polycaprolactone, and hydroxyapatite for shell design and ICA-loaded chitosan microspheres for the core, and showed great potential for bone regeneration and repair of tibia bone defects in rabbit models [101].

Zhu et al. (2022) [102] designed a novel injectable and thermoresponsive ICA-loaded composite hydrogel made by in situ crosslinking of hyaluronic acid, and Poloxamer 407, as a 3D cell culture system for intra-articular injection of bone marrow mesenchymal stem cells in a rat model of OA. This injectable hydrogel showed good biocompatibility in chondrocyte and mesenchymal stem cell cultures, promoted the proliferation and differentiation of mesenchymal stem cells through the Wnt/β-catenin signaling pathway, and relieved pain by regulating the expression of the anti-inflammatory cytokine IL-10 and collagenase-3 (MMP-13) in the OA model [102].

## 5. Conclusions and Future Prospects

Osteochondral tissue engineering based on hydrogels, stem cells, and signaling molecules showed considerable potential for the repair of the complex osteochondral structure. Due to its chondroinductive, osteoinductive, anti-inflammatory, and non-toxic effects, ICA could be used as a key constituent within the composition of composite hydrogels intended for osteochondral tissue repair, in order to improve the chondrogenesis and osteogenesis processes inside the osteochondral defects. At present, few studies have been conducted on ICA embedding in micro- and nano-formulations to increase its bioavailability and potential activity. Future studies are needed to address these key issues, along with more in-depth analyses of ICA interactions and mechanisms of action.

## Figures and Tables

**Table 1:** Composite hydrogels and scaffolds loaded with ICA for osteochondral defects repair.

Composite Hydrogels and Scaffolds	Experimental Model	Results	References
ICA-alginate hydrogel 3D complexes	in vivo	Enhanced articular cartilage repair in a mouse osteochondral defect model by improving the ICRS II histological score, compared to controls	[87]
ICA-hydroxyapatite/COL hydrogel	in vitro	Upregulated expression of chondrogenic and osteogenic genes (RUNX2, alkaline phosphatase, osteocalcin) and enhanced matrix synthesis of glycosaminoglycans and COL type II;	[56,96,97]
	in vivo	Higher expression of COL types X (marker of calcified layer formation), II (in neo-cartilage layer), and I (in new subchondral bone)	
ICA-functionalized nanodiamonds	in vitro	Increased osteogenic markers secretion (alkaline phosphatase, calcium) and mRNA level (alkaline phosphatase, COL type I, osteopontin, RUNX2);	[93]
	in vivo	Bone regeneration by the upregulated expression level of osteogenic marker genes (alkaline phosphatase, RUNX2, osteocalcin); inhibited osteoclast activity	
ICA/β-tricalcium phosphate disks	in vitro	Promoted proliferation and differentiation of Ros17/28 cells; no effect on attachment and morphology of Ros17/28cells; bone-apatite formation on the surface of disks after 3 days of soaking in simulated body fluid solution	[90]
	in vivo	Enhanced the bioactivity of β-tricalcium phosphate; new bone formation with fibrous tissue and slight inflammatory reaction	
ICA-calcium phosphate cement tablets	in vitro	Enhanced in vitro osteogenic differentiation	[73]
	in vivo	Accelerated bone regeneration at 4 and 6 weeks after transplantation	
ICA-self-crosslinked network functionalized with Sr-doped biphasic calcium phosphate bioceramics	in vitro	Co-delivery system with potential synergistic effect on promoting osteogenesis by an increased level of osteogenesis-related proteins alkaline phosphatase, osteocalcin, and BMP2	[81]
	in vivo	Inhibited osteoclastogenesis	
ICA-Chitosan/hydroxyapatite	in vitro	Cell compatibility	[92]
	in vivo	Promoted osteogenic differentiation of human bone marrow stem cells, osteoconduction, and osteoinduction	
45S5 bioactive glass doped with ICA and gelatin-coating	in vitro	Hydroxyapatite formation in simulated body fluid after 14 days of immersion	[95]
ICA-releasing PCL/PLGA/nanohydroxyapatite 3D printed composite scaffold	in vitro	Promoted osteogenic differentiation of MC3T3-E1 cells	[98]
	in vivo	Healing of calvaria bone	
